# An Uncommon Tooth Fusion of Mandibular Primary Lateral Incisor with Canine

**DOI:** 10.3390/clinpract11010016

**Published:** 2021-02-21

**Authors:** Saleh Ali AlKlayb, Darshan Devang Divakar

**Affiliations:** 1 Pediatric Dentistry, Ministry of Health, P.O. Box 11134, Riyadh 11433, Saudi Arabia; dr.saleh.alklayb@gmail.com; 2 Dental Health Department (Oral Medicine and Radiology), College of Applied Medical Sciences, King Saud University, P.O. Box 10219, Riyadh 11433, Saudi Arabia

**Keywords:** canine, fusion, lateral incisor

## Abstract

Fusion is a congenital disturbance affecting primary dentition mostly during morpho differentiation of the primary tooth germs. Fusion leads to the union of two or more primary teeth by the enamel and dentin while the pulp and roots remain separate. These abnormalities may be unilateral or bilateral. Prompt diagnosis and a treatment plan in such anomalies may help to overcome problems concerning aesthetics, caries susceptibility and space management. This report describes a case of unilateral fusion of the primary mandibular lateral incisor and canine and aims to evaluate any associated pathology.

## 1. Introduction

Primary and permanent dentition are affected by various types of dental anomalies which could be developmental or induced. In the primary dentition, the most frequent anomaly we come across in clinical practice is fusion among primary teeth [1]. Dental fusion is defined as the partial or complete union, during development, of two or more adjacent teeth [2]. It occurs when two separate tooth germs fuse and form a single large-sized tooth which has two separate roots and crown united by enamel and dentin [3]. It has been suggested that the most probable etiology for the formation of fused teeth is a physical action between the tooth buds undergoing morph differentiation due to which they come near. As a result of the pressure, there is necrosis of the intervening tissue, which causes the enamel organ and dental papilla to fuse [4]. Fusion has X-linked familial inheritance [5]. The prevalence of fusion in primary dentition is 0.5 to 2.5% and is much lesser in permanent teeth [6]. Tooth fusion may be either complete or incomplete, unilateral or bilateral [7]. Sometimes, two separate pulp chambers and root canals are observed. Nevertheless, fusion can also occur between a regular tooth bud and a supernumerary tooth germ. In these cases, the number of teeth is fewer if the anomalous tooth is counted as one tooth [8]. In germination, tooth bud division is partial, which leads to a large crown that has one root and one canal. Gemination and fusion are predominant in primary dentition, with incisors being more affected.

## 2. Case Report

**Figure 1:**
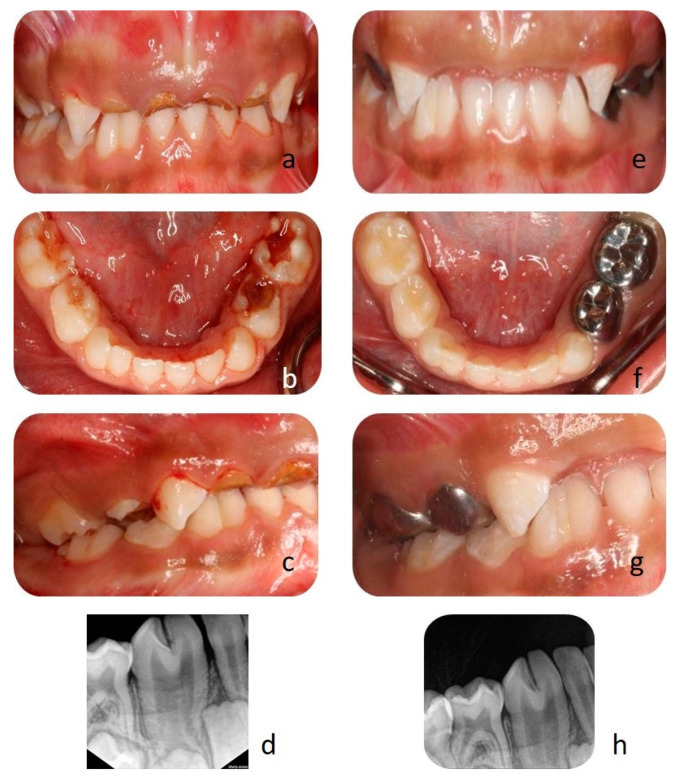
(**a**,**b**) Deep dental caries involving the pulp of primary upper central and lateral incisors and molars. (**c**) Fusion of canine and lateral incisors separated by a deep groove, (**d**) radiograph of fused teeth. (**e**) Post extraction. (**f**,**g**) Pulpotomy with stainless steel crowns, tooth-colored fillings on molars. (**h**) Fissure sealant between the groove of the fused tooth.

A four-year-old girl was reported with a chief complaint of dental pain and swelling in the upper anterior teeth for three months, with no other significant medical history. Intraoral examination revealed dental caries involving all primary molars and primary upper central and lateral incisors (Figure 1a,b). Interestingly there was an unusual clinical finding with mandibular right lateral incisor and canine seen as an enlarged bifid crown, i.e., 83 and 82 with deep labio-lingual groove (Figure 1a–c). A comprehensive intraoral radiograph was advised for most carious teeth, and also a radiograph was taken for the bifid crown, which revealed complete tooth fusion with fused pulp chambers and root canals (Figure 1d).

Due to patient’s negative behavior, clinical findings of multiple dental issues, full mouth rehabilitation under general anesthesia was considered. Extraction of hopeless primary teeth was performed, as was pulpotomy with stainless steel crowns for deep caries lesions. Tooth colored fillings for initial carious lesions was carried out (Figure 1e–g). A fissure sealant was applied between the deep labio-lingual grooves of fused teeth as a preventive measure (Figure 1h). The patient was followed for 2-week, 3-month, 6-month and 1-year post-treatment for evaluation, oral hygiene and topical fluoride application.

## 3. Discussion

The union of two separate tooth germs at the stage of teeth development or at the time of union leads to a developmental anomaly called tooth fusion. Fusion is caused by the physical forces between two or more teeth germs before calcification. The inner enamel epithelium and the dental papilla of the adjacent teeth fuse and later calcify while the pulp chamber and roots remain separate [9]. Certain factors governing fusion are racial and genetic factors [10], Anguilo et al. [11], classified fused teeth based on their morphology and extent of fusion as follows:

Type I: bifid crown, single root;
Type II: large crown, large root;
Type III: two fused crowns, double conical root;
Type IV: two fused crowns, two fused roots.

In the present case, a unilateral fusion between the right primary lateral incisor and canine was observed in a 4-year-old girl. This observation holds good with other studies reported since fusion in primary dentition are usually unilateral [12]. However, in some cases, bilateral fusion was also observed as reported by Gupta et al. [7,13]. According to the literature, a large tooth with incisal notching, labial or palatal grooving, or radiographic evidence of a separate or fused pulp chamber, or root, was recorded in southern Chinese and Mongoloid population studies in permanent dentition [14,15,16,17,18,19,20] but fusion of unilateral canine and lateral incisors in primary dentition have not been reported in any cases in Saudi Arabia. Furthermore, a double tooth is any tooth-like structure which resembles two complete, or partially complete teeth.

From the above classification, our case could be classified as Type III. Type III cases are found to be most commonly occurring compared to other types and are also most affected by dental caries along with type IV fusion [21,22].

Fusion in primary teeth has been associated with developmental malformations in the succeeding permanent teeth. It has been reported that the permanent teeth that replaced the fused teeth were either congenitally missing or double teeth or peg-shaped incisors [23]. Thus, careful clinical monitoring is necessary in these cases. Fusion is commonly associated with primary dentition. Since Type III fusion is more commonly associated with dental caries, appropriate preventive measures must be taken, and regular clinical observation must be carried out so that the permanent teeth are not affected.

## Data Availability

No new data were created or analyzed in this study. Data sharing is not applicable to this article.

## References

[1] Hagman F. (1985). Fused primary teeth: A documented familial report of case. ASDC J. Dent. Child..

[2] Tasa G.L., Lukacs J.R. (2001). The prevalence and expression of primary double teeth in western India. J. Dent. Child..

[3] Tomizawa M., Shimizu A., Hayashi S., Noda T. (2002). Bilateral maxillary fused primary incisors accompanied by succedaneous supernumerary teeth: Report of a case. Int. J. Paediatr. Dent..

[4] Uÿs H., Morris D. (2005). ‘Double’ Teeth—A Diagnostic Conundrum. Dent. Update.

[5] Golabi M., Ito M., Hall B.D., Opitz J.M. (1984). A new X-linked multiple congenital anomalies/mental retardation syndrome. Am. J. Med. Genet..

[6] Schuurs A., Van Loveren C. (2000). Double teeth: Review of the literature. ASDC J. Dent. Child..

[7] Gupta T., Manuja N. (2015). Bilateral fusion of primary mandibular incisors: A rare case report. J. Clin. Diagn. Res. JCDR.

[8] Zhu M., Liu C., Ren S., Lin Z., Miao L., Sun W. (2015). Fusion of a supernumerary tooth to right mandibular second molar: A case report and literature review. Int. J. Clin. Exp. Med..

[9] Chunawalla Y.K., Bijle M.N.A. (2011). Pulp therapy in Maxillary fused primary central and lateral incisor: A Case Report. Int. J. Contemp. Dent..

[10] Duncan W.K., Helpin M.L. (1987). Bilateral fusion and gemination: A literature analysis and case report. Oral Surg. Oral Med. Oral Pathol..

[11] Aguilo L., Gandia J., Cibrian R., Catala M. (1999). Primary double teeth. A retrospective clinical study of their morphological characteristics and associated anomalies. Int. J. Paediatr. Dent..

[12] Brook A., Winter G. (1970). Double teeth. A retrospective study of ‘geminated’ and ‘fused’ teeth in children. Br. Dent. J..

[13] Kapur R., Kapur R., Gupta R., Kapur R. (2011). Bilateral mandibular fusion in primary dentition—A case report. Indian J. Dent..

[14] Walker R.T. (1988). Root form and canal anatomy of mandibular second molars in a southern Chinese population. J. Endod..

[15] Walker R.T. (1987). Root form and canal anatomy of maxillary first premolars in a southern Chinese population. Dent. Traumatol..

[16] King N.M., Tongkoom S., Wong H. (2010). Morphological and numerical characteristics of the Southern Chinese dentitions. Part III: Anomalies in the primary dentition. Open Anthropol. J..

[17] King N.M., Tsai J.S., Wong H. (2010). Morphological and numerical characteristics of the southern chinese dentitions. Part II: Traits in the permanent dentition. Open Anthropol. J..

[18] King N.M., Tsai J.S., Wong H. (2010). Morphological and numerical characteristics of the southern Chinese dentitions. Part I: Anomalies in the permanent dentition. Open Anthropol. J..

[19] King N.M., Tongkoom S., Wong H. (2010). Morphological and numerical characteristics of the southern Chinese dentitions. Part IV: Traits in the primary dentition. Open Anthropol. J..

[20] Kamminga J., Wright R. (1988). The Upper Cave at Zhoukoudian and the origins of the Mongoloids. J. Hum. Evol..

[21] Açıkel H., İbiş S., Tunç E.Ş. (2018). Primary fused teeth and findings in permanent dentition. Med Princ. Pract..

[22] Zengin A., Celenk P., Gunduz K., Canger M. (2014). Primary double teeth and their effect on permanent successors. Eur. J. Paediatr. Dent..

[23] Yuen S., Chan J., Wei S. (1987). Double primary teeth and their relationship with the permanent successors: A radiographic study of 376 cases. Pediatric Dent..

